# Physiology and Chemistry of Old Age

**Published:** 1870-05

**Authors:** S. P. Cutler

**Affiliations:** Holly Springs, Miss.


### Selections.

Physiology and Chemistry of Old Age.—By S. P. Cutler, M. D., Holly Springs, Miss., *Read before the American Medical Association, New Orleans.*—That slow but sure and gradual process that sweeps and undermines all animated nature, may be regarded as a normal or physiological, not pathological or diseased action in a strict sense.

There is probably a probationary stage in man and animal, or a period of greater or lesser duration in different individuals, when decay and reproduction exactly balance each other, which marks a given period in each individuality, before which time the reproductive, after which decay predominates.

It is right here a question, whether the rapidity of development or growth in anywise governs the process of future decay, or is in any way coincident with physiological decline? We have good reason to believe that after the close of this stationary stage, that the organism gradually becomes mineralized, or, as it were, fossilized, by the cells becoming clogged with unoxidizable matter; in consequence, lowering cell activity and lessening vital heat and force or energy, and in proportion the *vis vitæ.*

There is a gradual filling up of cells with life antagonistics, gradually crowding life out of the least vital first, then the more vital, until ultimately, by this stealthy encroachment, life is driven out by displacement. The above statements may be strikingly illustrated by the fact that the meat of old animals is less nutritious, unless made suddenly fat from a lean condition. The meat of all young animals is more juicy than that of old. Young and tender, old and tough, is a very old adage.

Why will not a given amount of carbon and hydrogen as ternary food taken into the organism of old age produce the same amount of physical force or energy as in youth or middle age, digestion being equal in both cases? If the same amount of food be equally digested in both cases, then absorbed into the circulation and carried into the tissues and cells, and then oxidized in the same time, the same amount of physical force would be developed in each case.

We may admit that the same amount of carbon and hydrogen is taken in a given time in each and digested, still, this does not prove the fact that this food is oxidized in the same ratio after entering the tissues in both cases; admitting that the same amount of oxygen enters the organism in both cases, does not prove the fact that this oxygen is all consumed in both cases equally, as a portion may pass out in the one case un-oxidized, in the other, not in the form of debris, as also might a certain portion of the food pass out after undergoing normal digestion, as debris.

The food entering the tissues is not proof that it all entered the cells where the greatest amount of oxidation takes place. If, then, a portion of the ingested food enters the portals of the tissues without entering into the sanctum sanctorum or inner chambers of the tissues, it may pass out again unchanged by the reversal of the forces that carried it in, that is, by a change of its polarity and becoming dead, instead of living matter, so far as its relations to the organism at the time is concerned. It might be asked why the same amount of ingested food is not permitted to enter the cells in both cases?

The only answer I can give at present, is that the cells themselves are already partially filled with unoxidizable materials or mineralized matter; thereby mechanically excluding a portion of the nutritive elements. Under such circumstances, this additional quantity of food, more than can be consumed, must act as a clog, obstruct and weaken the animal forces; as a certain amount of force is required to carry in and out this useless load which can be of no service whatever, on the contrary, an injury, having a tendency to produce plethora, gout and other diseases.

It might be asked, why the cells become clogged with mineralized matter as age advances? Could we give the answer, the mystery of old age, if not life itself, would be solved.

It is extremely difficult to find access to cell structures so as to recognize changes taking place during life, even if such a thing were possible, their size being so small, it would be perhaps an impossibility to distinguish any actual difference in the character of cell contents. This could be determined by chemical analysis.

There are certain tissues that may be made the basis of comparison at different stages of life, that may serve to throw much light on this recondite subject. I allude to the hard tissues or bones, the most accessible being the dental structures, as they can be readily procured, even from the living subject, at all ages.

We find these organs when first ruptured, with a larger hollow or canal, having a greater per cent. of cartilage or dentine, than in middle life, and more in middle than in old age. Whether there is actually a probationary or stand-still stage in these and other hard structures, is not yet settled; the probability is, that there is a slow but constant change steadily going on.

There certainly must be a period or culminating stage when the fullest perfection and most favorable balance of destructive and reproductive elements is attained. This particular period might possibly be ascertained or approximated, and when these organs have remained healthy, might furnish an index to all other tissues of the body. After this period has been reached by these organs, there commences, and steadily progresses, a change in the anatomical constituents, which progresses pari-passu; no doubt with similar changes throughout the entire organism, until as before stated, life is entirely crowded out.

Death by old age, that slow but stealthy and certain process of crowding out of life from the organism, by filling up the cells with non vital, mineral elements, thereby not allowing room, if I may so express it, for life to continue its ceaseless roll. If this hypothesis be correct, we are gradually driven out of our earthly tabernacle by an enemy that never raises his siege. Some may, and do, hold out longer than others, though it is only a question of time. It may be inferred that nature or the organism gradually succumbs to antagonistic forces, acting both from within and without. When organic, or what are called chemico-vital forces, first counteract each other, physiology is sustained intact, and any deviation from these counterpoises converts physiology into pathology.

These counterbalances may be inferred to be decay and reproduction, oxidation and waste on the one hand and nutrition or reproduction on the other. Oxidation in the cells makes room for nutrition, otherwise no such thing could take place. Hypo and hyper nutrition and other causes may influence these conditions to a certain extent.

Why this condition from the highest endowments of organic life should be gradually undermined and destroyed by antagonistic forces so as to entirely break up the organization, has not yet been discovered or revealed any farther than the supposition that each architypal, primordial cell may have received its impress of limitation from the Creator, subject to limited modifications from certain modifying circumstances.

The greater the amount of normal oxidation taking place in the organism, the greater the amount of vital force of such organism. Energy of nutrition just neutralizing such oxidation, the resultant being *a tertium quid*, called life force or energy. It is, in other words, the oxidizing and breaking up of organic elements of the organism, and the conversion of it into organic elements that develops life force—life being motion, and oxidation being the cause of such motion, the bent spring, the falling weight, the dynamical battery. To return to the hard tissues. The teeth, like bones, continue to ossify inwards from childhood to old age, until in extreme cases all life ceases and the tooth becomes dead from occlusion of pulp canal and tubuli, with great preponderance, of earthly salts over animal elements, and becomes friable and brittle. All other bones may be supposed to be in a similar condition, more ossified, less vital, more brittle and friable, the consequence would be a cooling of these tissues to such an extent as to seriously chill the sarcode and fluids, and ultimately causing death, from cooling down below a certain life-sustaining point.

As the organs cool, vital energy ebbs pari passu, unless sustained by artificial stimulants, such as alcohol and certain others, a portion of which passes the rounds of the circulation unchanged, and by its direct agency on the blood and vessels, additional vigor is imparted so long as under its influence. Some of the alcohol may be burned as combustible food, also favoring oxidation in the cells generally, so long as under its influence.

Life itself could not be prolonged to any considerable extent by any such artificial means, but their proper use would be of service; in fact, would be essential in old age, as excitors of vital forces.

As the food of old persons varies but little from that of youth, there must be an excess of unappropriated lime salts retained in the organism an indefinite time—which, the osseous system failing to appropriate, seems to clog the system by finding its way into the cells, more especially cartilage and all similar tissue cells first, but subsequently into more vital structures, shutting out oxidation or all activity as well as mechanically obstructing life, energy or force.

After a given amount of arrestation of cell activity, life itself sinks below the life-sustaining point by both cooling and mechanically obstructing as in cases of calcification of the valves of the heart and other important structures.

The question here arises, is there any known or unknown means of arresting this mineralization of tissues, especially in the bones of advancing age, as other tissues *a priori* calcify only after the hard tissues fail to make farther use of the lime salts taken with food in the form of normal nutrition.

Other tissues harden in a given ratio, which may vary in different individuals, and in the same individual under different circumstances.

Can there be any means devised that will retard this mineralizing process in any way? Possibly there can. It is evident that if lime-water is used for drink and food, known to contain a large per cent. of this salt, that there are at least more favorable opportunities afforded favoring this process. I might here make two suggestions: First, to use food and water known to be the most free from phosphates. Secondly, to make use of food containing a large per cent. of vegetable acids, such as fruits, also vegetables in their green or fresh state, and vinegar largely, during meals. Eggs and milk are known to contain all the minerals in excess of almost all other kinds of food; butter may be used freely, also fat meats, both forming combustible food chiefly. Sugar, lemons, oranges, apples, cherries, plums, peaches, currants and many other fruits and berries, are good. The rinds or peelings of fruit should always be discarded in advancing age, as they contain the chief minerals. In young subjects this order should be reversed, as they need an excess of minerals up to the time of full development and growth.

All acids containing hydrogen, as most vegetables do, decompose phosphates and carbonates, forming soluble salts, which could not be deposited permanently in the cells, either of bones or other tissues.

Any free lime salts floating in the system, or even in the cells, may be decomposed and more salts formed and washed out of the organism. Not only this, but even the bones themselves may be made to give up any surplus portion of lime in this way in old age, so as to render them less dense and friable, and more susceptible of nutrition, and old age even put farther off indefinitely.

The object of vegetable acids, as diet, would be to establish an acid diathesis, similar to the disease called rickets in children, which depends on such a diathesis that removes lime from the bones, and in some cases, in very young children, prevents even the deposit of lime in certain bones and portions of bones. Mellitis osseum in adults, especially females, after puberty, and pregnant women, necessarily depends on a morbid acid diathesis, unlike the former disease. I do not propose to carry the acid to such an extent as to develop a diseased condition of the bones or any part of the organism, only to use sufficient to prevent a too rapid accumulation of lime in the system. Normal waste of bones can take place from no other cause, save that of acid action, dissolving out the lime salts, while oxygen alone removes the gelatine or cartilage, similar to any other soft or fleshy tissue. Any vegetable acid containing hydrogen, as above stated, will decompose both phosphate and carbonate of lime, either in or out of the organism, if sufficiently concentrated.

I do not propose medication as a prophylactic or preventive of old age, but only suggest proper dietetic regulations, in order to prevent premature old age. This much I believe to be possible.

Bone decay or waste depends on a double or dual process, namely, that of acid removing lime, and oxygen removing the osteine similar to any tissue which gives place to nutrition, which can follow after waste only. In this respect bone nutrition is unlike any other. The exhalation of all other tissues is the work of oxygen only. Decay and reproduction, old tissues disintegrated and new protoplasm added is the order of life, death and regeneration; die that we may live. Without death, there can be no life, so said Bichat, who first propounded this great fundamental truth.

All decay is chemical action, acting both in harmony and in cpposition to vital action, and both essential to vital existence; both acting in concerted antagonisms. All chemical action depends on molecular physics, and all life manifestations are based on molecular polarity or dynamics similar to that of chemical. Again, the food of children should contain an excess of lime salts, also that of pregnant females. The food of advancing age should contain the smallest possible amount of lime salt, and the largest amount of vegetable acids. Females who have borne the most children, are frequently the longest lived, gestation imparting additional vital energy for the time being, by setting up new energies in her system ; at the same time, drawing heavily on the lime salts of the food for the new growth or development of osseous structures. At the same time, there is generally set up in the enciente female an acid diathesis, which acts heavily on the bones, this is made apparent by dental decay during this period. The above facts go to sustain my hypothesis or theory.

Excess of food or overeating, even of the proper kind, is injurious in advancing age ; serving to over-distend the cells, causing undue deposits of minerals, which clog the system and check vital energy, and to a greater extent and more permanently in the bones than in the sarcode.

All the tissues in old age become less oxidizable and less vital, even the fat in the cells, owing to loss of water and excess of carbon. Gray hairs show and wrinkled skin indicate loss of vital energy ; in fact the whole system becomes yellow and less fair in advancing age.

In short, the system becomes mineralized and carbonized and mummyfied. The bone cells first become clogged and cooled, causing a heavy dragging sensation as if the bones were becoming heavy and cold. This is actually the case, the earth in the bones increases, consequently they become less vital and nutritious. Fragilia, Osseum Senilis the result.

How to remedy the loss of water in the soft tissues and excess of carbon, is not so easily determined. One fact is observable, as people grow old they drink less water, also sleep less, the tissues become dryer and less juicy. A very old truism, young and tender, old and tough.

The most of old animals are tougher, dryer and less nutritious, unless they are rapidly fattened from the lean state, as before stated.

Carbonaceous matters and minerals predominate in old persons. The predominance of carbon may depend on evaporation of water or its elements in advanced age.

Wood of any kind, when kept dry, loses elements of water, and continues to do so for centuries, turns yellow and becomes brittle. The same may be said of all organic substances, either in the living or dead state. These are significant facts.

Mineralizing of cell contents and carbonizing of cell walls or structures, seems to be the only constitutional causes of decay and old age. Some may die of old age, ten, twenty or more years sooner than others. Some may live to be one hundred and fifty or more years, and even then not die of old age.

As I have already stated in a published article on physiology, all heat and vital force depends exclusively on oxidation, but the probable mode of such oxidation and per cent. of different oxidizable elements of the various proximate and elementary principles, do not at present concern us.

All life forces may be regarded as the sum or aggregate of all the forces of the different elements that enter into organisms as pre-existent, only acting in new directions and under different circumstances, the new endowments being the result of new combinations not found to exist outside of organic life. In new endowments of matter only, are new directions of forces exalted or lifted up.

Finally, who shall say, what is really the outside limits of life under different circumstances? Who shall gainsay that man's organism may not be so retained in its youthful condition as to furnish boys and girls at one hundred years, by scientific classification of food for the human family?

From recent researches of Darwin, Spencer and others, what may we not hope for, if the human family can but receive a share of the scientific labors of such men in relation to longevity as set forth in the above article?

Removal to warmer climates in advancing age, where more light and heat are absorbed, may add to the years, other things being equal. In a recent article on food, by Leibeg, no distinction is made, nor is any reference made to the opinions of many of the eminent authors in relation to longevity.

